# Different paradigms of transcranial electrical stimulation improve motor function impairment and striatum tissue injuries in the collagenase-induced intracerebral hemorrhage rat model

**DOI:** 10.1186/s12868-022-00689-w

**Published:** 2022-01-29

**Authors:** Amir Reza Heidarzadegan, Asadollah Zarifkar, Narges Sotoudeh, Mohammad Reza Namavar, Amir Hossein Zarifkar

**Affiliations:** 1 Department of Physiology, School of Medicine, Shiraz University of Medical Sciences, Shiraz, Iran; 2 Shiraz Neuroscience Research Center, Shiraz University of Medical Sciences, Shiraz, Iran; 3 Histomorphometry and Stereology Research Centre, Shiraz University of Medical Sciences, Shiraz, Iran; 4 Department of Anatomical Sciences, School of Medicine, Shiraz University of Medical Sciences, Shiraz, Iran; 5 Clinical Neurology Research Center, Shiraz University of Medical Sciences, Shiraz, Iran

**Keywords:** Intracerebral hemorrhage, Striatum, Transcranial electrical stimulation, Motor function, Stereology

## Abstract

**Background:**

In the horizon of therapeutic restrictions in intracerebral hemorrhage (ICH), recently, non-invasive transcranial electrical stimulation (tES) has achieved considerable prosperities. Translational studies have postulated that transcranial direct current stimulation (tDCS) and the other types of tES remain potentially a novel therapeutic option to reverse or stabilize cognitive and motor impairments.

**Objective:**

The aim of this study was to comparatively evaluate the effects of the four main paradigms of tES, including tDCS, transcranial alternating (tACS), pulsed (tPCS), and random noise (tRNS) stimulations on collagenase-induced sensorimotor impairments and striatum tissue damage in male rats.

**Methods:**

To induce ICH, 0.5 μl of collagenase was injected into the right striatum of male Sprague Dawley rats. One day after surgery, tES, was applied to the animals for seven consecutive days. Motor functions were appraised by neurological deficit score, rotarod, and wire hanging tests on the day before surgery and postoperative days 3, 7, and 14. After behavioral tests, brain tissue was prepared appropriately to perform the stereological evaluations.

**Results:**

The results indicated that the application of the four tES paradigms (tDCS, tACS, tRNS, and tPCS) significantly reversed motor disorders in collagenase-induced ICH groups. Further, the motor function improvement of tACS and tRNS receiving rats in wire-hanging and rotarod tests were higher than the other two tES receiving groups. Structural changes and stereological assessments also confirmed the results of behavioral functions.

**Conclusion:**

Our findings suggest that in addition to tDCS application in the treatment of ICH, other tES paradigms, especially tACS and tRNS may be considered as add-on therapeutic strategies in stroke.

## Introduction

Stroke is one of the principal causes of death and disability worldwide. Stroke categorizes into the ischemic and hemorrhagic types, and ischemic represents the most common of cases (87%) [1]. Bleeding into the brain tissue by the rupture of vessels causes intracerebral hemorrhage (ICH). Prognoses of this type of stroke rely on many factors including the initial clinical presentation, rapidity of diagnosis, and time to initiation of intervention. Hypertension [2, 3] and anticoagulation therapy [1] remain the most principal causes of hemorrhagic stroke.

Since the treatment of hemorrhagic stroke includes some limits for using drugs, recently, novel therapeutic methods, like non-invasive stimulation of the brain through transcranial electrical stimulation (tES) have been presented. By itself, the induction of neuroplastic changes by tES has added momentum over the recent years [4]. In the previous study, we found that different paradigms of tES prevent Aβ-induced cognitive impairment in the novel object recognition test [5]. The stimulation is easily carried out by employing direct current (DC) over the scalp [6] without any invasive techniques. The earlier study has shown this weak transcranial stimulation can induce long-term and polarity-specific changes in the excitability of the cerebral cortex in humans [7]. In this technique, two electrodes are typically placed on two precise areas of the scalp to complete the flow circuit [8]. The locations of the electrodes are essential to control the orientation and spatial distribution of the current and finally the effectiveness of the intervention [9]. This method is indicated to provide valuable effects in the treatment of nervous system disorders like depression, anxiety, chronic pain, and Parkinson's and Alzheimer’s diseases in addition to the course of rehabilitation in cognitive impairments [10].

The four main transcranial paradigms of tES include direct current (tDCS), alternating current (tACS), random noise (tRNS), and pulsed current (tPCS) stimulations.

tDCS is the most widespread technique in neurotransmission modulation, and its effects rely on the active electrode polarity to persuade cortical excitability changes. In general, the polarity-dependent mechanisms are recognized to cause membrane depolarization by anodal stimulation which increases cerebral excitability, or membrane hyperpolarization by cathodal stimulation leading to a decrease in neuronal excitability. Also, excitability changes depend on the neuronal geometry and axodendritic axis of the neurons [11].

Mechanisms of the tDCS have been investigated more than the other three tES paradigms. Clinical studies have proposed potential therapeutic properties of tDCS and it is effective in the treatment of numerous disorders, including post-stroke motor disorder [12], aphasia after stroke [13], epilepsy [14], chronic pain [15], and Parkinson's disease [16].

The tACS is another paradigm of tES wherein biphasic alternating electrical pulses are applied to modify neuronal activities. In contrast to tDCS which exerts inhibitory effects due to polarity, the effects of tACS are affected by the current frequency and independent of the polarization of the electrodes. The tACS does not change neuronal excitability but drags the neuronal firing from a substantial number of underlying neurons to the exogenous frequency. The polarization of neurons shows the current exerted to it, resulting in a sinusoidal fluctuation of the membrane potential. Since this fluctuation is frequency-dependent and linearly relative to the applied current, lower-frequency stimulation persuades larger polarization than higher frequencies [17, 18].

The tRNS is a special type of tACS that includes the application of random noise oscillations above specific brain regions to modulate cortical plasticity. One of the suggested mechanisms of tRNS is the increase of neuronal excitability via random resonance, whereby weak neural signal detection in the central nervous system is amplified when noise is added. The advantages of this new technique, compared with tDCS, include the absence of sensitivity to the polarity of the electrodes and the reduction of skin sensitivity to the electrodes during stimulation. In most previous studies, a spectrum of frequencies between 1 to 640 Hz has been used. In fact, the current generated by the noise stream practically is in the range of 1 mA [5, 18].

The tPCS represents a discontinued direct current stimulation with a constant amplitude. In this paradigm, the stimulation is interrupted at regular intervals, and the definitions of pulse duration, frequency, and inter-pulse intervals are added to the current. It has also been shown that tPCS retains potential benefits for cognitive functions [5, 19].

Compared to the other three paradigms, the tDCS is the most studied and its mechanisms have been further investigated. In addition to the evidence on clinical benefits in some diseases, using tDCS in healthy people can also improve memory and other cognitive functions [20, 21]. Our recent study indicated that tDCS enhances athletic performance outcomes of experienced bodybuilders [22]. However, the precise pathways involved in tDCS effects are not fully discovered and further studies are necessary for its routine clinical use. It is believed that the application of an electric field with sufficient strength and time can cause a fast increase in the electrical conductivity of cell membranes. This may be the result of an increase in permeability for ions and molecules. Nonetheless, knowledge about the effects of neurotransmission, neurochemical markers, neuronal pathways, or neuronal interactions is not quite precisely understood.

The proposed mechanisms of action of tDCS on the brain include the changes in the neuronal activity, cerebral blood flow, and the osmotic activity of the brain as well as brain functional communication patterns, synaptic and non-synaptic effects, and neurotransmitter modulation [23]. In this way, tDCS might be potentially considered as a suitable add-on treatment for improving motor functions in the stroke based on the pathophysiology of this disease.

The number of animal studies that are employing this technique to explore the mechanisms of tDCS is increasing [24]. Yu and colleagues revealed that tDCS application after the onset of cognitive dysfunction leads to some beneficial effects on motor behavior in Alzheimer’s disease. They also found that anodal tDCS improved spatial memory in this disorder [25].

Many studies have addressed the effects of tDCS in patients with stroke [26–28]. Some studies have also shown the improvement effects of tDCS as a combination therapy on motor function in these patients [29–31]. A recent study showed that repetitive tDCS improves functional motor and somatosensory outcomes during the first month of stroke [32]. In 2019, Bai et al. also stated that tDCS is effective for the recovery of the motor dysfunctions in the upper and lower limbs of stroke patients [33].

Considering the impairing effects of ICH on cognitive and motor functions and suggested neuroprotective results of tES on the other hand, this study was designed to comparatively evaluate the effects of different tES paradigms on motor behavior impairment and striatum tissue damage induced by collagenase-induced ICH in rats and finally to determine which of the tES paradigms is more effective in this regard.

## Materials and methods

### Animals

Adult male Sprague−Dawley rats (250−280 g) were used. Animals were maintained at room temperature (23 ± 2 °C) under a standard 12–12 h light-dark cycle with lights on at 7:00 A.M. Food and water were available ad libitum. The experimental protocols were approved by the Ethics Committee of Shiraz University of Medical Sciences (SUMS, IR.SUMS.REC.1397.039) and animal care was according to the NIH Guide for the care and use of laboratory animals. Fifty-six rats were randomly divided into the seven groups (n = 8 per group) including the control, sham, ICH, ICH+ anodal tDCS, ICH + tACS, ICH+ tRNS and ICH+ tPCS groups. In the control group, neither surgery nor intervention was performed. In the sham group, after the surgery and injection of the vehicle (PBS), electrodes were placed but no electrical stimulation was performed. In the ICH group, ICH was induced but rats did not receive tES. ICH+tDCS, ICH+tACS, ICH+tRNS, and ICH+tPCS groups, after induction of ICH, received tDCS, tACS, tRNS, and tPCS, respectively.

### Surgical procedure

The animals were anesthetized with an intraperitoneal injection of mixed ketamine (100 mg/kg) and xylazine (10 mg/kg) and then, they were placed in a stereotaxic frame for ICH surgery. A 2-cm incision was made in the shaven head skin to remove all soft tissues on the skull before drilling. According to Paxinos and Watson [34], a 26-G Hamilton needle (10 μl, 700 series, Hamilton Company, Switzerland) was placed in the right striatum in the coordinates of 0.36 mm anterior (AP), 3 mm lateral (ML), and 5.4 mm ventral (DV) to the Bregma [35]. After collagenase micro-injection (0.5 μl), acrylic dental cement was used to seal the drilled hole, and the skin was sutured. Meloxicam (0.1 mg/kg) was injected to maintain postoperative analgesia. To apply electrical stimulation, a plastic tube (inner diameter: 2 mm) was placed on the right frontal cortex (on the skull surface) for the electrode. The plastic tube was anchored to the skull employing stainless screws and acrylic cement [5].

### Collagenase injection

We used collagenase VII from clostridium histolycum (Sigma-Aldrich, USA) high purity, purified by chromatography, Type VII, ≥ 4 FALGPA units/mg solid, lyophilized powder, 1,000–3,000 CDU/mg solid (CDU = Collagen digestion units). Collagenase (0.5 μl of 0.15 U/μl in PBS) was injected through the Hamilton needle unilaterally into the right striatum gently within 5 min. The sterilized PBS (vehicle) was replaced in the sham group [36].

### Induction of electrical stimulation

The plastic tube which was placed on the skull surface on the surgery day was filled with a sponge and saline. Rats were covered with a towel, and the electrodes were inserted. In all rats, the anode electrode was placed into the plastic tube above the right frontal cortex and the cathode electrode was positioned onto the ventral thorax with a corset. Current flows from the anode electrode to the cathode electrode. For reducing the contact impedance, sponges were moistened with saline before electrical stimulation [5]. tES was started the day after surgery. In all tES groups, stimulations were applied to the awake and freely moving rats for seven consecutive days, 20 min per session, with current intensities of 200 μA, and was ramped for 10 s. In the tACS group, the frequency of stimulation was 30 Hz, in the tPCS group, the pulse length and inter-pulse intervals were 13 msec and 20 msec, respectively and in the tRNS group, the random frequency of stimulation was between 1 and 200 Hz [5, 37]. In the sham group, electrodes were placed, but no stimulation was applied. On days 3, 7, and 14 after surgery, neurological deficit scores were determined and then rotarod and wire hanging tests were performed for each rat.

### Neurological deficit score (NDS)

All six parameters of the NDS, including body symmetry, climbing, gait, circling behavior, compulsory circling, and front limb symmetry, were measured on the day before surgery and days 3, 7, and 14 of post-ICH for each rat. Each item was scored from zero (severe deficit) to three (normal) and the total scores with 3 referring to the maximum deficit and 18 to normal [38].

### Wire hanging test

For assessment of the wire-hanging test, a wire (2 mm × 60 cm) was installed between two platforms with a height of 50 cm, and then the animals were placed midway for a maximum of 5 min. In this way, the grip strength and balance were observed on the day before surgery and days 3, 7, and 14 post-ICH. To prevent any injury caused by possible falls, a pillow was placed on the ground. The suspension time of the rat on the wire was recorded [39, 40].

### Rotarod test

The rotarod is widely used to evaluate motor coordination in rodents. The rotating speed of the rotarod cylinder represented four revolutions per minute (rpm) at the beginning of the test, after which the speed was accelerated to 40 rpm over the course of four min. The latency to falling of animals was measured. Rats were tested three times, and the results were averaged. For adaptation, they were instructed for three days before surgery. Rats were placed horizontally on the cylinder of the apparatus (ATF CO, Iran). In this orientation, with turning on the system, animals have been rotated. The purpose of this test was to determine how the rats could maintain themselves [41].

### Tissue processing

Fourteen days after surgery, rats were anesthetized and perfused transcardially with a cold 0.09% saline, followed by 4% buffered paraformaldehyde. Brains were instantly removed and post-fixed in the same fixative overnight at 4 °C, transferred to 30% sucrose (Sigma, St. Louis, MO, USA) in phosphate-buffered saline for 48 h; then frozen and stored at 20 °C until the further process. The brains were sectioned serially and coronally, using a cryostat (Leica, Germany) at a thickness of 40 μm throughout the brain and were immersed into a 12-well plate containing cryoprotectant solution and kept in a 20 °C freezer, until needed. Every 18th section (at an interval of 720 μm) was slide-mounted and stained with Cresyl violet [42, 43].

### Stereological evaluations

The volume of the right hemisphere and striatum and hemorrhage in the right striatum were unbiasedly estimated by the means of the point-counting method, using Cavalieri’s principle. Randomly superimposing lattices of test points on sectional images generated by systematic random sampling and counting those points within specified tissue compartments [44, 45].

The total number of neurons, non-neurons, and dead cells were estimated in the right striatum using the optical disector technique. Focusing on optical sections for a known distance developing z-dimension of the disector volume leads to enumerate of particle number based on the unbiased sampling frame. The unbiased sampling frame is considered an unbiased sampling volume with both upper and lower optical sections leading to the exclusion planes in three dimensions [44].

### Differentiation of neurons from the non-neurons

Before estimating the number of neurons and non-neurons, it had to specify the criterion of differentiating the neurons from non-neurons, including glial cells. For this purpose, these specifications are considered:

1. Clear and plentiful cytoplasm with Nissl bodies in the cell body of the neuron
2. Existence of cell processes in the nerve cell
3. Existence of one clear nucleolus in neurons and lack of it in non-neurons
4. Euchromatin substance in the big nuclei of neurons versus the heterochromatin masses in the small nuclei of non-neurons [46, 47]. It is necessary to say that using the optical disector and oil immersion lens with a wide numerical aperture (NA = 1.4) provides the power of good recognition of the non-neurons from the small neurons.

### Method of differentiation and criteria of the live and dead cells

By light microscopy, necrotic or apoptotic neurons can be identified as exhibiting pyknosis, karyorrhexis, karyolysis, and cytoplasmic eosinophilia [48, 49]. It should be emphasized that with usual Cresyl violet staining, it is not definitely possible to say the dead neuron has been undergone necrosis or apoptosis. For this reason, the terminology of the dead cell has been used in this study. Furthermore, we used the non-neuron terminology that includes glial and endothelial cells in the Cresyl violet in which differentiation between these cells is not clearly possible.

### Data analysis

All behavioral tests and decoding were performed blindly. Data for each group were analyzed using GraphPad Prism version 6.00 for Windows, (GraphPad Software, La Jolla, CA, USA) and described using graphs, means, and standard error of the mean. The Kolmogorov–Smirnov test was administered to determine the normal distribution of data, and the two-way ANOVA with the Tukey–Kramer post hoc test was used to fulfill the intergroup comparison. Repeated-measure ANOVA was used to assess the time effect for behavioral tests. In all tests, the statistical significance level was P < 0.05.

## Results

There were no differences between the body weights of groups before and 14 days after surgery (data not shown).

### Behavioral assessment tests

#### Neurological deficit score test

**Fig. 1:**
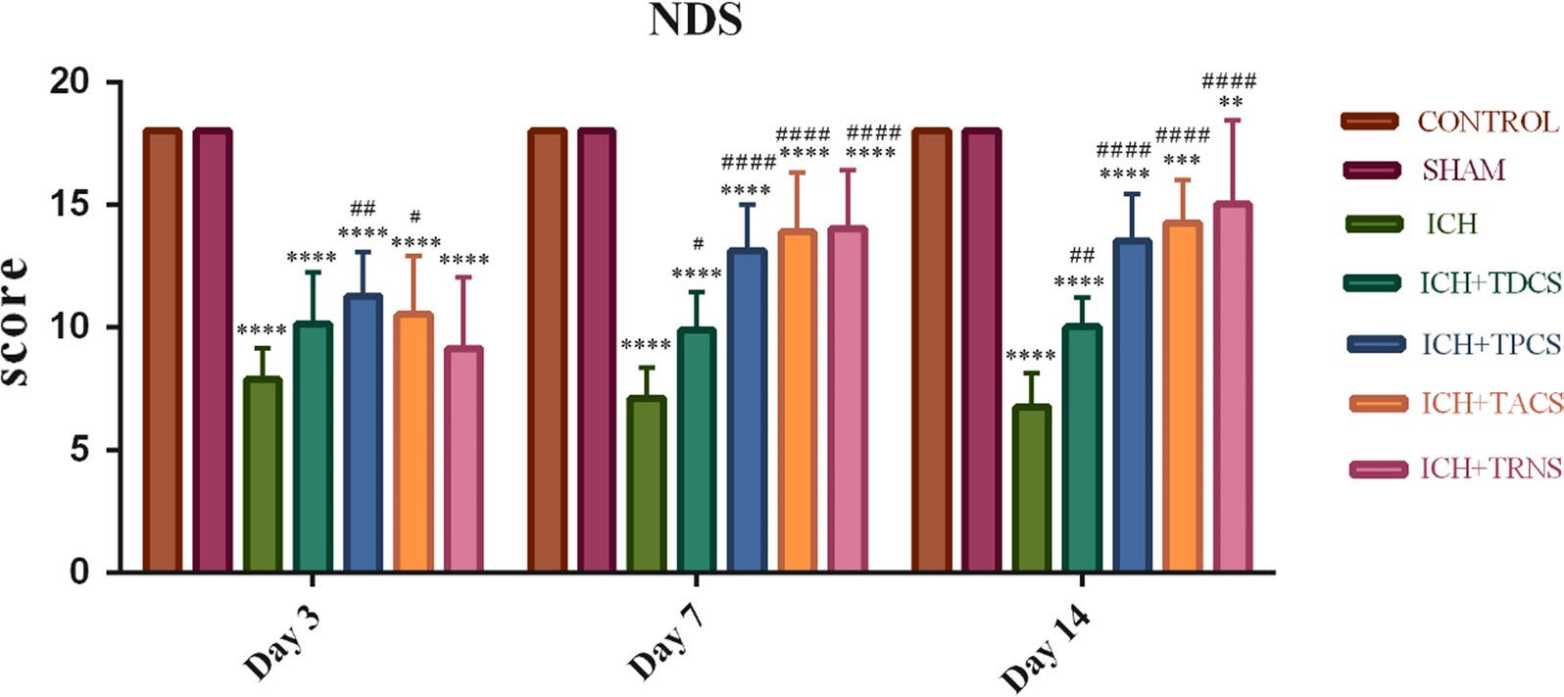
The tES paradigms effect on NDS induced by ICH. Data are presented as mean ± SEM (n = 8). ^**^ P < 0.01, ^***^ P < 0.001, ^****^ P < 0.0001 vs. the sham group and ^#^ P < 0.05, ^##^ P < 0.01, ^####^ P < 0.0001 vs. the ICH group. There was no significant difference between the control and sham groups. ICH, intracerebral hemorrhage; NDS, neurological deficit score; TACS; transcranial alternating current stimulation; TDCS, transcranial direct current stimulation; tES, transcranial electrical stimulation; TPCS, transcranial pulsed current stimulation; TRNS, transcranial random noise stimulation

We measured the neurological deficit score (NDS) utilizing an 18-point scoring system in which lower scores mean more severe deficits. NDS in all rats in the sham and control groups was scored 18 in 1 day before surgery, and 3, 7, and 14 days after the surgery (Fig. 1). None of the rats showed neurological deficits prior to surgery and there was no significant difference between control and sham groups (data on the day before surgery were not shown). The NDS was significantly reduced on the 3rd day in the ICH group and all ICH-tES groups compared with the sham group (P < 0.0001, Fig. 1). On days 7th and 14th, NDS of ICH-tES groups were significantly higher than of ICH group scores (tDCS = P < 0.05, tPCS = P < 0.0001, tACS = P < 0.0001, tRNS = P < 0.0001). Furthermore, there was a significant difference in this score among the studied groups in terms of time effect (P = 0.0004). Two-way ANOVA multiple comparisons of NDS did not show any significant differences between tES-treated rats on day 3 of evaluation. However, on the 7th and 14th days, there was a significant difference between the tDCS-treated group and tPCS, tACS, and tRNS groups (P < 0.01). This difference was more appreciable on the 14th day.

#### Wire hanging test

**Fig. 2:**
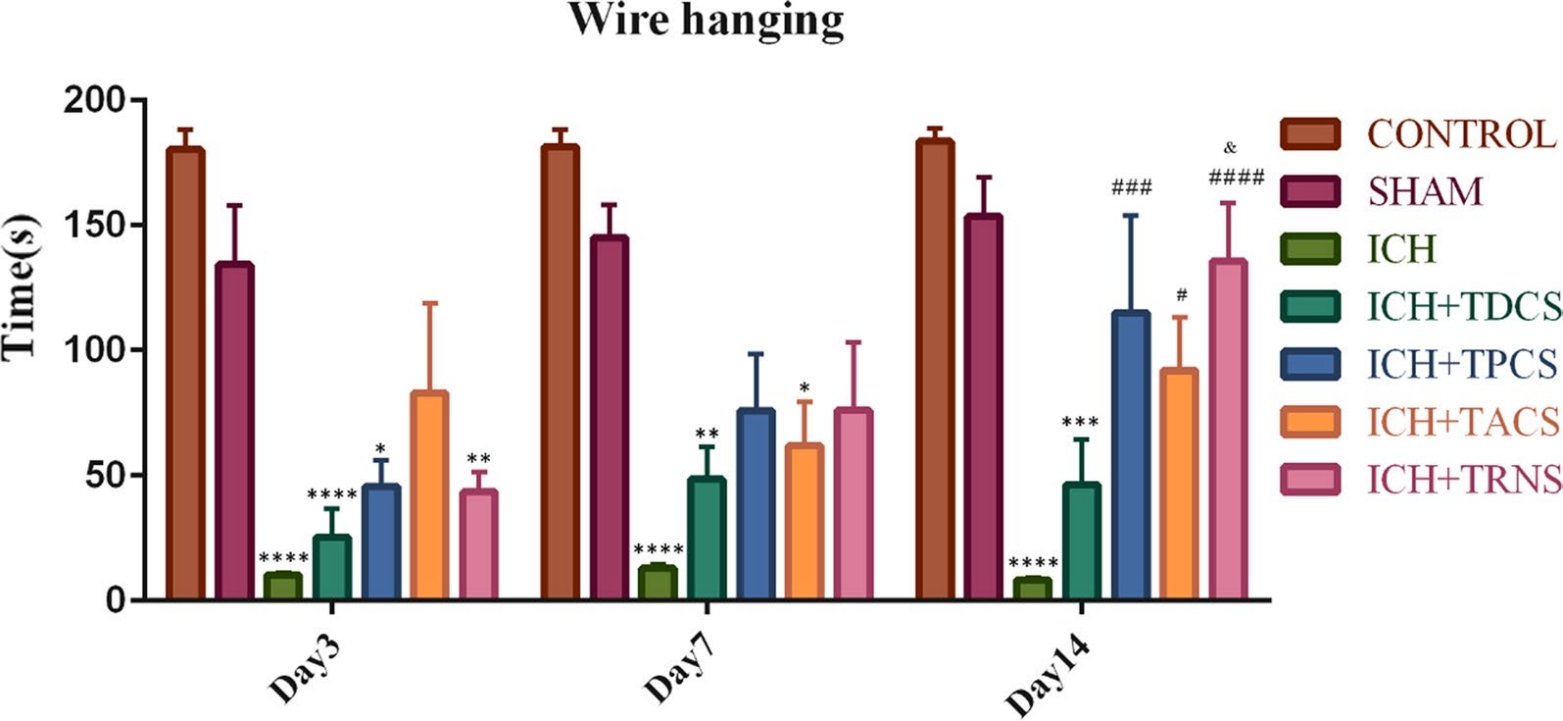
The tES effect on locomotor deficits evaluated by wire hanging test (falling latency). Data are indicated as mean ± SEM (n = 8). ^*^ P < 0.05, ^**^ P < 0.01, ^***^ P < 0.001, ^****^ P < 0.0001 vs. the sham group and ^#^ P < 0.05, ^###^ P < 0.001, ^####^ P < 0.0001 vs. the ICH group and ^&^ P < 0.05 vs. the TDCS group. There was no significant difference between the control and sham groups. ICH, intracerebral hemorrhage; TACS; transcranial alternating current stimulation; TDCS, transcranial direct current stimulation; tES, transcranial electrical stimulation; TPCS, transcranial pulsed current stimulation; TRNS, transcranial random noise stimulation

The wire-hanging test was indicating the grasping for forelimb strength and motor coordination. In this test, the suspension time of the rats which remained on the wire with their forelimbs was recorded. ICH caused a significant decrease in the falling latency compared with the control and sham-operated groups (Fig. 2). Based on our results, falling latency significantly increased from the 3rd to 14th days in ICH-tES rats (P < 0.05) and this increase was more obvious in the tRNS group at 14th days (Fig. 2). There was a significant difference in the suspension time among the considered groups in terms of the time effect (P = 0.0385). Comparison of falling latency between tES-treated groups did not show significant differences on days 3 and 7. However, just the tRNS-treated group had better suspension time in comparison with tDCS (P = 0.0014).

#### Latency to fall off rotarod

**Fig. 3:**
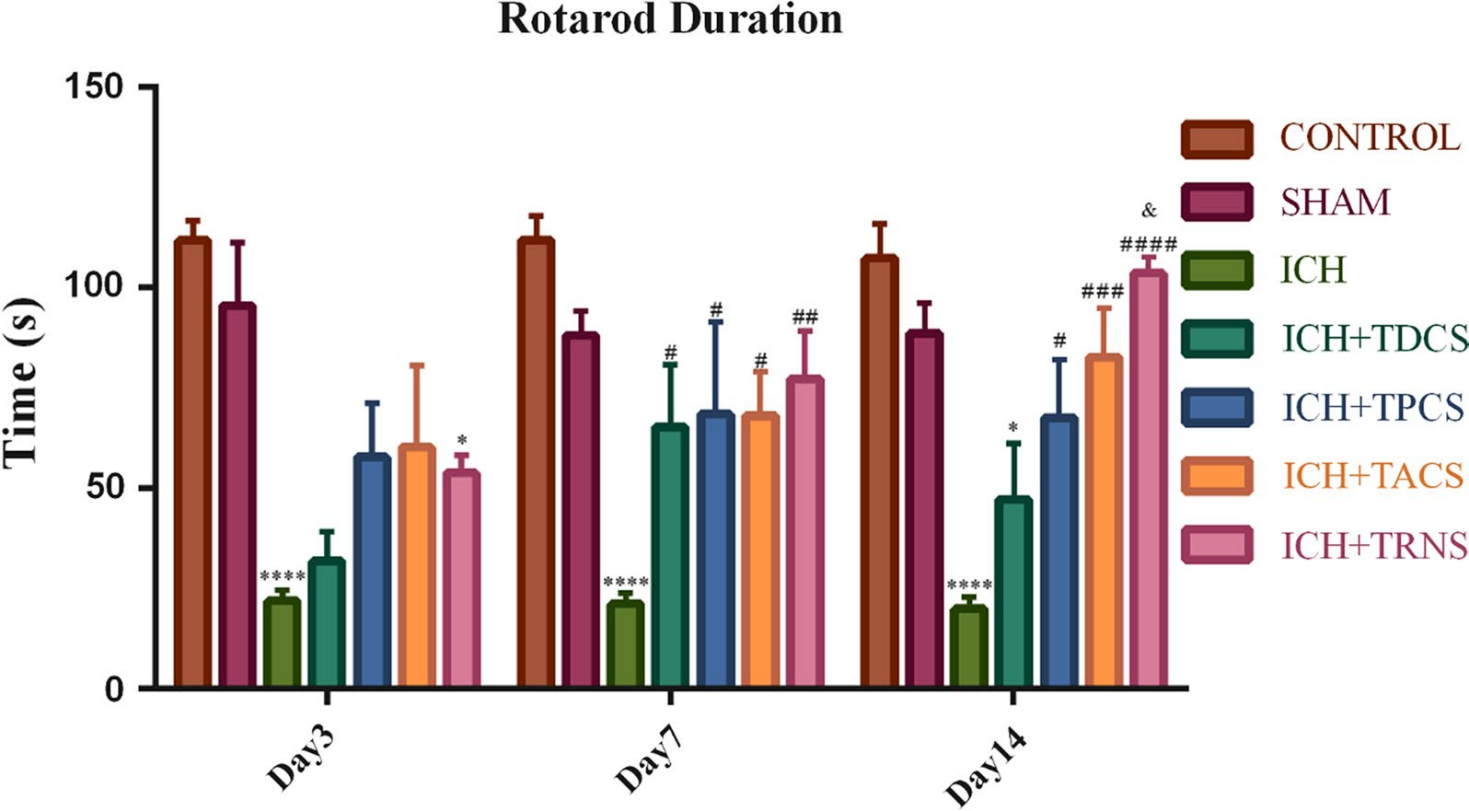
The tES effect on locomotor deficits evaluated by the rotarod test (latency to fall off). Data are displayed as mean ± SEM (n = 8). ^*^ P < 0.05, ^****^ P < 0.0001 vs. the sham group and ^#^ P < 0.05, ^##^ P < 0.01, ^###^ P < 0.001, ^####^ P < 0.0001 vs. the ICH group and ^&^ P < 0.05 vs. the TDCS group. There was no significant difference between the control and sham groups. ICH, intracerebral hemorrhage; TACS; transcranial alternating current stimulation; TDCS, transcranial direct current stimulation; tES, transcranial electrical stimulation; TPCS, transcranial pulsed current stimulation; TRNS, transcranial random noise stimulation

The duration of the rotarod test of all groups is indicated in Fig. 3. The results showed there was not any significant difference between the control and sham groups in one day before surgery (data not shown) and 3, 7, and 14 days after surgery. ICH in the striatum significantly decreased this parameter in the 3rd, 7th, and 14th days after surgery when compared with the sham group. tES treatment increased this time from the third day after surgery, however, this increase was statistically significant from the 7th day and the maximum effect was observed in the tRNS group (Fig. 3). Repeated-measure ANOVA did not show any significant difference among the planned groups in terms of time effect (P = 0.1780). Two-way ANOVA multiple comparisons of the rotarod test did not show any significant differences between tES-treated rats on the third and seventh day of evaluation. Similar to the wire hanging test, the only tRNS-treated group had a better time in comparison with the tDCS-treated group (P = 0.0102).

### Histological and stereological results

**Fig. 4:**
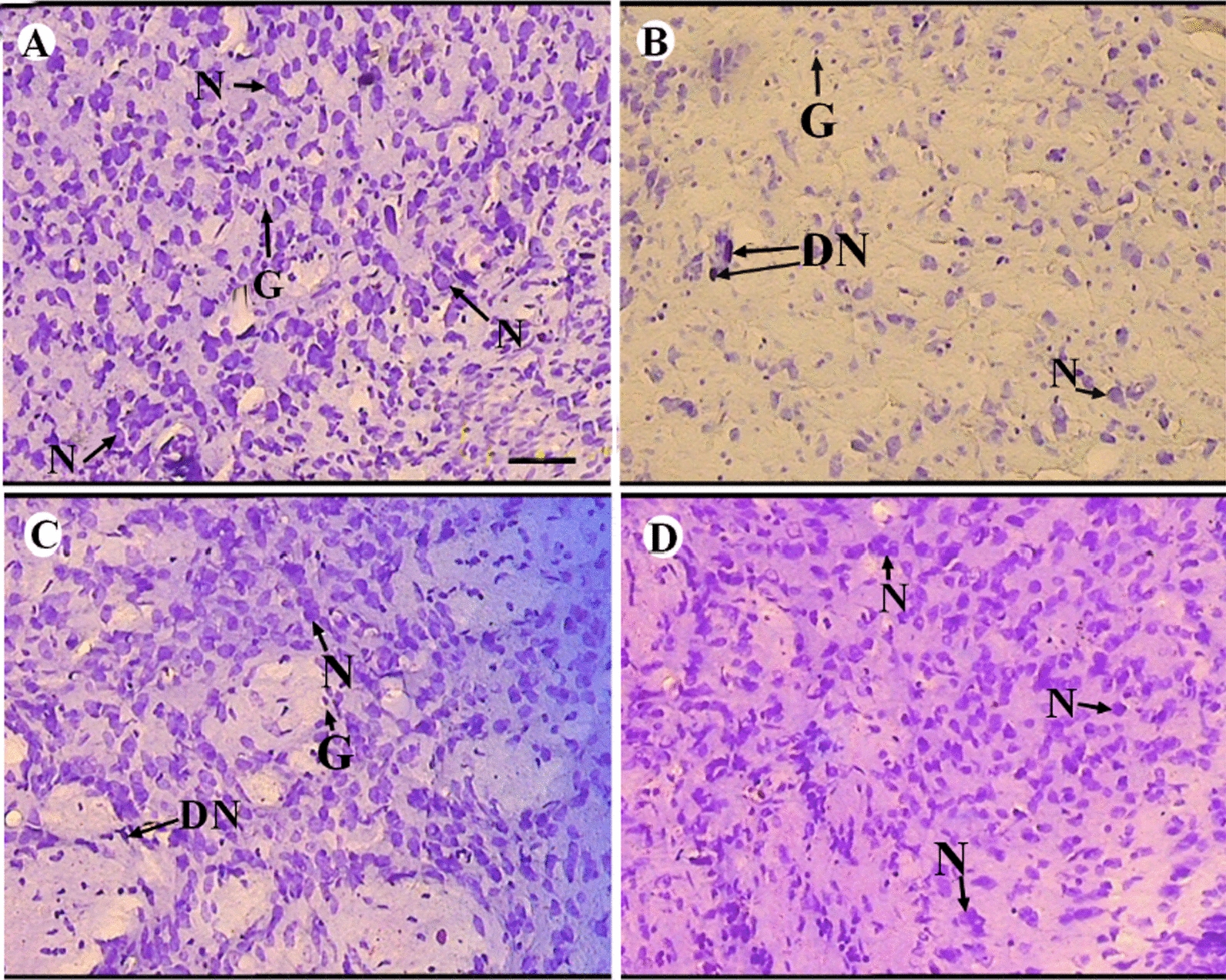
Representative photograph of the right striatum histology stained with Cresyl violet. **A** Normal neuron (N) and non-neuronal cells (G) with normal spatial distribution are observed in the sham group. **B** Intracerebral hemorrhage (ICH) showed dead cell (DN) and altered the cell spatial arrangement (ICH group). Treatment of ICH with transcranial electrical stimulation preserved tissue changes in its normal arrangement (transcranial direct current stimulation, (**C**) and this improvement was more obvious in the transcranial random noise stimulation group (**D**). Scale bar = 50 µm

Histological evaluation of the right striatum in the sham and control groups showed normal nervous tissue with normal neurons and non-neurons that represent a normal spatial arrangement and numerical density of neuron and non-neuron cells and there was no dead cell in these groups (Fig. 4A). The collagenase-induced intracerebral hemorrhage model in this study showed hemorrhage in the striatum. Furthermore, ICH in the striatum showed dead cells and changed the spatial arrangement and density of cells (Fig. 4B). tES paradigms almost returned striatum to its normal status or at least it prevented severe tissue changes and the tRNS paradigm exerted a better effect (Fig. 4C and D).

#### The total volume of the right hemisphere

**Fig. 5:**
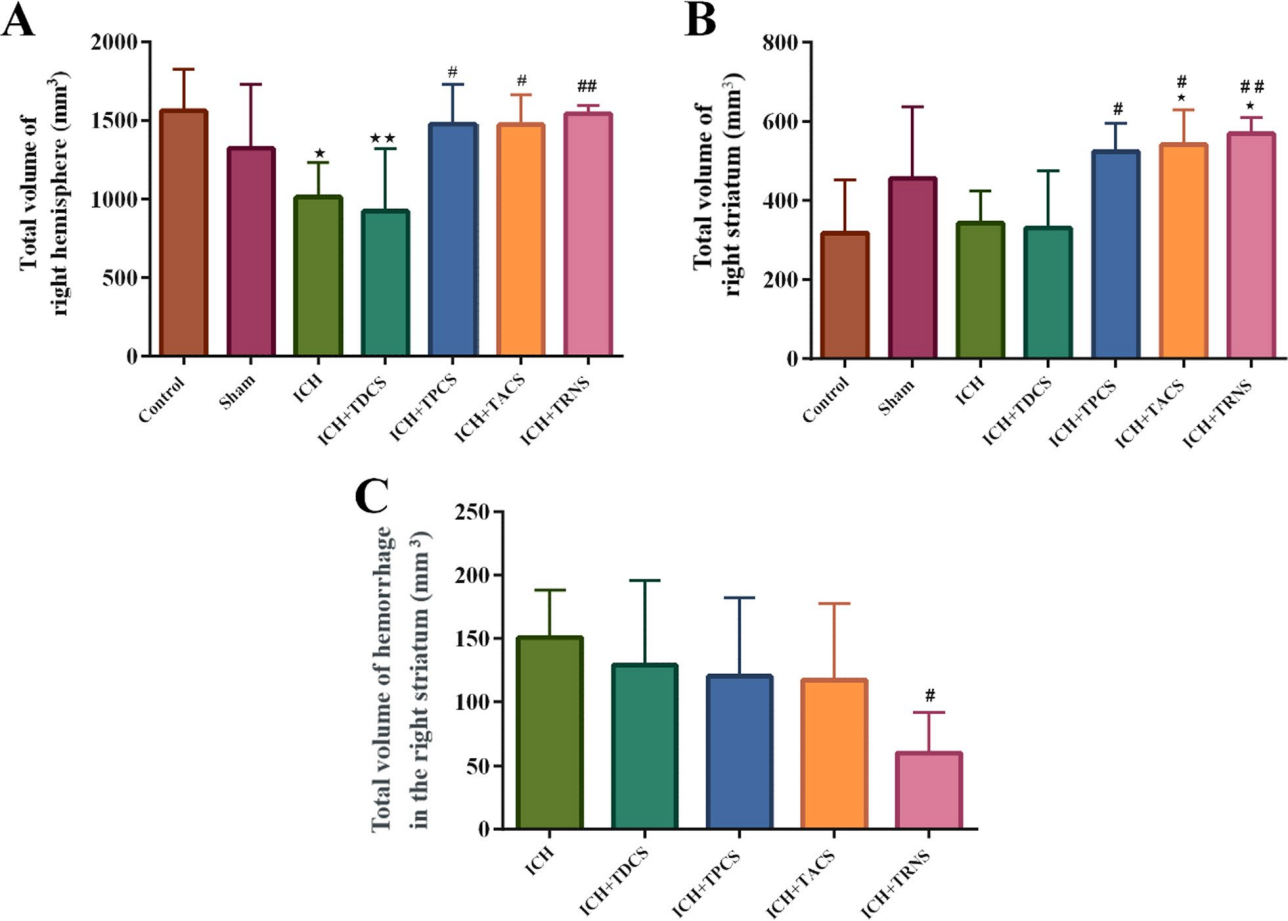
**A** The total volume of the right hemisphere in different groups. **B** The total volume of the right striatum in investigated groups. **C** The total volume of hemorrhage in the right striatum in the ICH and ICH-treated groups. Data are mean ± SEM (n = 8). ^*^ P < 0.05, ^**^ P < 0.01, vs. the sham group and ^#^ P < 0.05, ^##^ P < 0.01, vs. the ICH group. ICH, intracerebral hemorrhage; TACS, transcranial alternating current stimulation; TDCS, transcranial direct current stimulation; TPCS, transcranial pulsed current stimulation; TRNS, transcranial random noise stimulation

There was no significant difference between the volume of the right hemisphere in the control and sham groups (Fig. 5A). Intracerebral hemorrhage (ICH) significantly decreased the total volume of the right hemisphere in comparison with the control or sham groups (Fig. 5A). Although the tDCS paradigm did not exert a significant effect on the volume of the right hemisphere, other transcranial stimulation paradigms (tPCS, tACS, and tRNS) significantly increased this parameter when compared to the ICH group (P < 0.05, Fig. 5A).

#### The total volume of the right striatum

There was no significant difference between the volume of the right striatum in the control and sham groups. Infusion of collagenase into the right striatum did not significantly change this parameter, however, treatment with all paradigms of stimulation except the tDCS caused the increase of right striatum volume compared with ICH and this increase was even more obvious in the tRNS group (Fig. 5, tPCS and tACS = P < 0.05, tRNS = P < 0.01).

#### The total volume of hemorrhage in the right striatum

There was no observed hemorrhage in the striatum of the control and sham groups. Hemorrhage in the ICH groups was histologically obvious (Fig. 4B). Among the tES paradigms only tRNS decreased the hemorrhage in the striatum in comparison to the ICH group [P < 0.05, (and other paradigms had no significant effects) Fig. 5C].

#### The total number of neurons in the right striatum

**Fig. 6:**
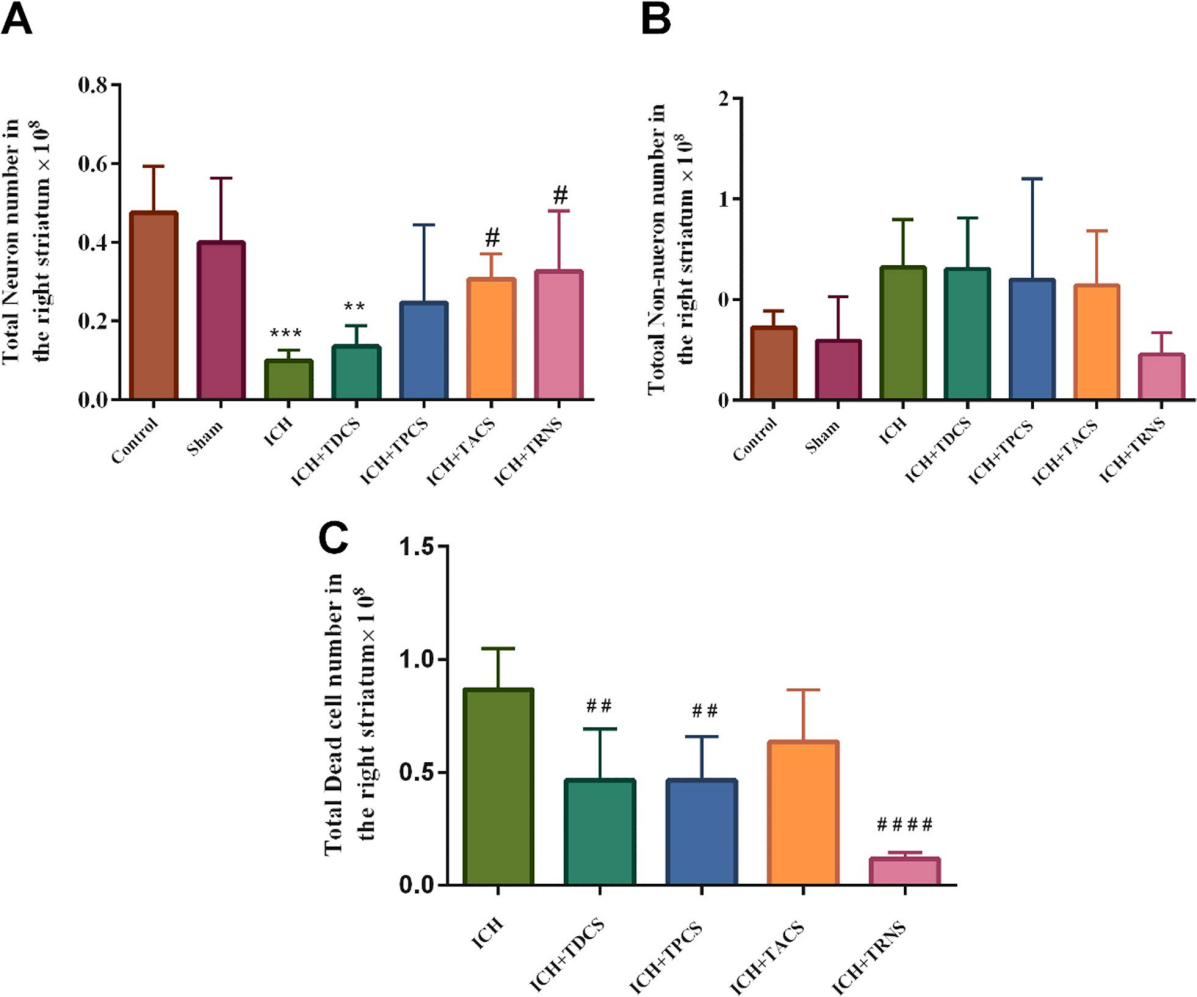
**A** The total number of neurons in the right striatum in different groups. **B** The number of non-neurons in the right striatum of considered groups. **C** The total number of dead cells in the right striatum of the ICH and ICH-treated groups. Data are shown as mean ± SEM (n = 8). ^**^ P < 0.01, ^***^ P < 0.001 vs. the sham group and ^#^ P < 0.05, ^##^ P < 0.01, ^####^ P < 0.0001 vs. the ICH group. ICH, intracerebral hemorrhage; TACS, transcranial alternating current stimulation; TDCS, transcranial direct current stimulation; TPCS, transcranial pulsed current stimulation; TRNS, transcranial random noise stimulation

Estimation of the neuron number in the striatum did not reveal any significant difference between the control and sham groups, but ICH in the striatum significantly reduced this parameter compared with the control group (P < 0.001, Figs. 4B and 6A). Treatment with tACS and tRNS significantly increased the number of neurons in the striatum (P < 0.05, Fig. 6A).

#### The total number of non-neurons in the right striatum:

There was not observed any significant difference in the number of non-neuronal cells in the striatum between groups. Although hemorrhage in the striatum partly increased these cells and tRNS treatment decreased this parameter, however, these changes were not substantial to produce statistically significant (Fig. 6B).

#### The total number of dead cells in the right striatum

Evaluation of striatum in the control and sham group did not show any dead cells, therefore, we did not include these groups for statistical analysis and comparison. Infusion of collagenase into the striatum indicated a very considerable increase in the number of dead cells in the striatum. All different paradigms of tES (except tPCS) significantly decreased the number of dead cells in the striatum (P < 0.01), however, tRNS exerted a magnificent effect on cell death (P < 0.0001, Fig. 6C).

## Discussion

Non-invasive brain stimulation techniques are widely used in the treatment of many disorders. In this study, we observed that different paradigms of tES application could ameliorate motor function impairments in ICH induced by collagenase administration into the striatum of rats. The collagenase-induced intracerebral hemorrhage model is a valuable and reproducible animal model for the study of the effects of hematoma and brain edema on the brain [50] and we adopted this suitable model in our investigation. In this model, the hemorrhage size is controllable which is induced by small vessel breakdown, and also can mimic the onset of spontaneous intraparenchymal hemorrhage and the expansion of continuous bleeding in ICH patients [51, 52]. Accordingly, in the present study, the ICH significantly reduced NDS, falling latency, and rotarod maintenance duration indicating expected damage had occurred in the striatum by collagenase action. Plus, the stereological finding revealed a reduced number of neurons and a substantial number of dead cells in the damaged striatum, which were correlated to the behavioral results.

In the current investigation, all applied tES paradigms exerted an improving effect on motor behaviors and tRNS produced the best effect. In our previous study, we found that tACS was more effective than the other three tES paradigms in memory tasks in a rat model of Alzheimer's disease [5]. It seems that electrical circuit activities of the hippocampus (involved in memory) and striatum (involved in motor function) are differently influenced by applied electrical current rhythms and frequencies.

Unlike tDCS, the other three stimulation patterns, it has not been studied much, and in this study, we examined the effect of three other tES paradigms on motor function. Previous studies have shown that tACS can modulate cortical excitability (EEG oscillations) and memory due to the relationship between brain oscillations and cognitive processes [53, 54]. In the present study, the effect of this paradigm on motor function was determined.

The prevailing hypothesis about the action of tACS is that alternating fields can increase or decrease the power of oscillatory rhythms in the brain, and in a frequency-dependent manner, through synchronizing and desynchronizing neuronal networks [17]. Abnormal brain rhythms are associated with pathologic conditions. Thus, the researchers are trying to treat these neurological diseases by modulating these brain rhythms, and the tACS paradigm with the application of a specific frequency creates this ability. This study could at least partly document the positive effects of tACS in that respect.

Laczo et al. reported that transcranial high-frequency tRNS increases human leg motor cortex excitability [55]. Likewise, our study showed the obvious effect of tRNS on the improvement of motor function in ICH rats. This effect can be related to modulating effects of tRNS on the oscillatory activity of the motor cortex [56]. However, to confirm that more studies and more accurate measurements should be done.

Our study was consistent with that of Inukai et al.that compared tDCS, tACS, and tRNS. Their findings showed that the tRNS was the most effective [56]. In the current study, the improvement effect of the tRNS paradigm on motor performance of ICH rats, assessed by rotarod and wire hanging tests, was more prominent than other paradigms. Further, the groups that received tES revealed better behavioral performance, and on the other hand, these groups were significantly different from the ICH group in terms of striatal volume and number of neurons. Therefore, it seems to be a correlation between behavioral function and the volume of the striatum, and the number of neurons. Meanwhile, in our study, tDCS in comparison with the other three paradigms had lesser effects on the time parameter in the rotarod and wire-hanging latency. Histological and stereological results in this study were in line with our behavior data regarding tDCS. In most previous studies, tDCS exerts improving effects, but in our investigation, these effects were not observed. This controversy might have been due to the number of stimulation sessions or the power of the electrical current to reach the underneath structures or perhaps due to the type of disorder or behavioral test studied. Nevertheless, tDCS produced a significant effect on the reduction of the number of dead cells in the right striatum (Fig. 6C).

Stereological results in the present study showed collagenase-induced ICH did not significantly change the volume of the right striatum, however, treatment with tES, increased this parameter even more than the control group (Fig. 6B). This can be attributed to both preventing cell death and stimulating neurogenesis in that structure by tES. The counting of dead cells and the total number of neurons in the striatum in this study confirm both hypotheses, although we have not directly evaluated the effect of tES on neurogenesis after ICH. However, few studies have shown that electrical stimulation can induce neurogenesis in animals [57–59] and therefore support the idea of using tES to induce regeneration and to promote recovery of function in neurodegenerative diseases.

Few stereological studies have reported the effect of electrical stimulation on the structural changes in the brain. However, in a previous study, Rueger et al. declared that applying tDCS after stroke may help to locally augment endogenous neural stem cells known to promote neuroprotection and repair [60].

It has been shown that stroke causes gliosis [57] and our data regarding evaluating non-neurons just show a slight increase in this parameter (Fig. 6B). ICH treatment with tES for one week could not significantly decrease these cells, although tRNS exerted a better effect. Nevertheless, the positive or negative role of gliosis in CNS repair remains to be elucidated [61].

Predominantly, it seems that behavioral results in accordance with histological findings show that almost all paradigms of tES retain benefits on the stroke, however, the tRNS paradigm is the best non-invasive therapeutic option for hemorrhagic stroke in the striatum. Undoubtedly, more studies are required to implement these treatments in clinical conditions.

However, the current study included several limitations: (1) cell death was not quantified by specific methods such as TUNEL; (2) neurogenesis that may be induced by tES, has not been evaluated by a specific technique; (3) we did not implement immunohistochemistry to differentiate neurons, glia, and endothelial cells in the striatum; (4) we are not sure regarding the long-term effects of tES treatment on the stroke, for this reason, it is recommended to evaluate behavioral and histological parameters for a long time after treatment. However, the experiments described here were primarily designed to focus on the effects of tES paradigms on the histological changes in addition to neurobehavioral functions.

## Conclusion

Based on our findings and those of the previous studies, the ameliorative effects of tES on the motor function improvement of collagenase-induced ICH in the striatum appears to be properly supported. Furthermore, the results of the current study showed that the use of multiple sessions of different paradigms of tES can reverse the motor impairment and striatum tissue injury induced by collagenase in a rat model; that tACS and especially tRNS paradigms are more effective in this regard. Based on such evidence, it could be expected that in addition to tDCS application in the treatment of ICH, other tES paradigms may be considered as add-on therapeutic strategies in stroke. More research is of course required to postulate such an impact in clinical settings.

## Data Availability

The datasets used and/or analyzed during the current study are available from the corresponding author on reasonable request.

## Abbreviations

DC: Direct current
ICH: Intracerebral hemorrhage
NDS: Neurological deficit score
SUMS: Shiraz University of Medical Sciences
tACS: Transcranial alternating current stimulation
tDCS: Transcranial direct current stimulation
tES: Transcranial electrical stimulation
tPCS: Transcranial pulsed current stimulation
tRNS: Transcranial random noise stimulation
